# Alteration of Metabolic Syndrome Is Associated with the Decreased Risk of Colorectal Cancer

**DOI:** 10.3390/jcm12154889

**Published:** 2023-07-25

**Authors:** Eun Hyo Jin, Yoon Jin Choi, Joo Hyun Lim, Cheol Min Shin, Kyungdo Han, Dong Ho Lee

**Affiliations:** 1 Department of Internal Medicine, Healthcare Research Institute, Seoul National University Hospital Healthcare System Gangnam Center, Seoul 06236, Republic of Korea; 65845@snuh.org (E.H.J.); limz00@gmail.com (J.H.L.); 2 Center for Gastric Cancer, National Cancer Center, Goyang-si 10408, Gyeonggi-do, Republic of Korea; 3 Department of Internal Medicine, Seoul National University Bundang Hospital, Seongnam-si 13620, Gyeonggi-do, Republic of Korea; scm6md@gmail.com; 4 Department of Biostatistics, College of Medicine, Soongsil University of Korea, Seoul 06978, Republic of Korea; hkd917@naver.com; 5 Department of Internal Medicine, Liver Research Institute, Seoul National University College of Medicine, Seoul 03080, Republic of Korea

**Keywords:** metabolic syndrome, colorectal cancer, abdominal obesity, glucose intolerance, high-density lipoprotein cholesterol

## Abstract

Metabolic syndrome (MetS) can be resolved through active control. We aimed to examine the effect of changes in MetS status on colorectal cancer (CRC) risk. A total of 5,704,611 Korean national insurance beneficiaries that received two consecutive biennial mandatory health exams (2009–2011) were followed-up until 2017. MetS was determined as the presence of at least three of five components. Participants were categorized into four groups according to the change in MetS status; MetS-never, -resolved, -developed, or -persistent. A Cox proportional hazards model adjusted for age, sex, smoking, alcohol drinking, and physical exercise was used. Participants who recovered from MetS had a higher risk of CRC than those free of MetS but had a lower risk than those with persistent MetS (HR: 0.91, 95% CI: 0.86–0.95 vs. HR: 0.75, 95% CI: 0.73–0.78; reference: persistence group). Among the five MetS components, resolving high blood pressure, abdominal obesity, and blood sugar had a preventive effect on CRC prevention, while normalization of lipid profile did not reduce CRC risk independently. Resolving MetS could reduce CRC risk compared to having persistent MetS, indicating the necessity of considering control of MetS as a CRC prevention policy.

## 1. Introduction

Colorectal cancer (CRC) is the third most common cancer and the second leading cause of cancer-related deaths worldwide [1]. Besides aging, the most powerful risk factor of CRC is a westernized lifestyle: more consumption of red meat than vegetables, a sedentary living pattern, and obesity can contribute to CRC development [2]. The above-mentioned unhealthy lifestyle causes obesity itself and metabolic syndrome (MetS), a group of conditions that can lead to heart disease, diabetes, and stroke [3]. At the same time, obesity is one component of MetS, and each reinforces the other; both are associated with an elevated CRC occurrence. Therefore, MetS has often been considered as a link between obesity and CRC [4].

Obesity has become a worldwide major public health problem, and in South Korea, the obesity rate has increased for about past 10 years, reaching 36.3% in 2019 [5]. During this period, South Korea has experienced a rapid increase in MetS, from 27.1% in 2001 to 33.2% in 2020 [6], being ranked second in the incidence of CRC worldwide in 2018, with 44.5 cases per 100,000 persons per year [7].

Fortunately, MetS can be prevented or reversed by making lifestyle changes, including weight reduction; exercising regularly; eating more fruits and vegetables, fiber-rich whole grains, and protein-rich legumes; and avoiding high-fat, high-salt, and high-sugar foods [8].

However, although several studies have reported a positive association between MetS and CRC development [9,10,11,12,13], whether changes in MetS status influence CRC risk has rarely been investigated. This could be of great clinical importance as it will not only suggest the relationship between MetS and CRC, but also provide evidence on whether escalating CRC risk could be reversed through the active control of MetS. Therefore, we aim to evaluate whether a change in the MetS status affects the risk of CRC using a Korean nationwide population-based dataset. In addition, we examine whether resolving individual MetS components or reducing the number of them could decrease the risk of CRC.

## 2. Materials and Methods

### 2.1. Data Source and Study Participants

The Self-report questionnaires provided by National Health Insurance Corporation of South Korea (NHIC) is the national insurer managed by the Korean government that covers about 97% of the Korean population. The NHIC recommends that its subscribers receive a standardized medical examination every two years. The database of NHIC (anthropometric measurements, laboratory tests, and standardized self-reported questionnaires) was utilized. This research was approved by the Institutional Review Board of the Seoul National University Bundang Hospital (X-1608/360-906). Every procedure concerning human participants was carried out in conformance with the 1964 Helsinki Declaration and its subsequent amendments or comparable ethical standards. This study used anonymized data; hence, the need for informed consent was waived.

### 2.2. Study Population

**Figure 1:**
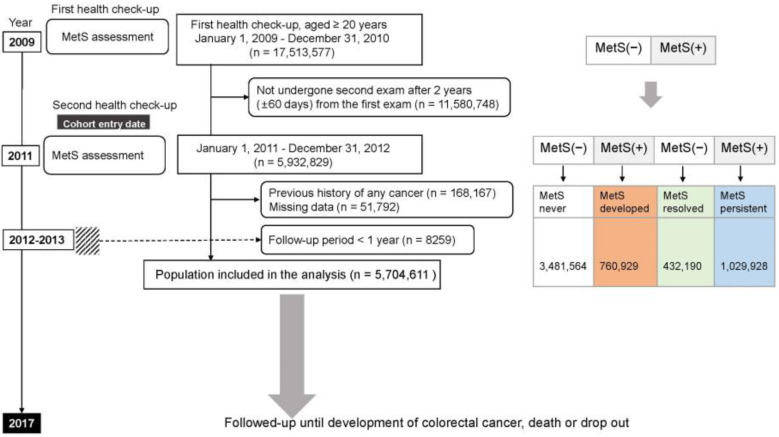
Flowchart of the enrollment process of the study cohort. The dashed line indicates those who were excluded because colorectal cancer was diagnosed within one year of study registration. MetS, metabolic syndrome; (+), yes; (−), no.

Among 17,513,577 individuals aged ≥20 years who underwent health screening between January 2009 and December 2010, we excluded 11,580,748 individuals who were not re-examined after 2 years (±60 days), those with a history of any malignancy at cohort entry (n = 168,167), and those with missing data (n = 51,792). Additionally, to clarify temporal relationships, enrollees who developed CRC and died within the first year of cohort enrollment were excluded (n = 8259). Ultimately, 5,704,611 individuals were analyzed and traced for the development of CRC, death, and loss of follow-up until 31 December 2017. The day of the second examination was set as a cohort entry: the information collected on that day was specified as a baseline characteristic. The participants were divided into four groups according to the four combinations of MetS status at the first and second exams; MetS-never, MetS-developed, MetS-persistent, or MetS-resolved groups. A schematic illustration of the study protocol is described in Figure 1.

### 2.3. Clinical Parameters and Biochemical Analyses

At the first (2009) and second examinations (2011), medical personnel measured the participant’s body mass index (BMI) and their systolic and diastolic blood pressures (mmHg). Blood samples were collected for the measurement of serum glucose, HDL cholesterol, LDL cholesterol, and triglyceride levels after fasting for at least 8 h. Standardized, self-report questionnaires were used to collect data: age (years), sex, alcohol consumption, and smoking status (Supplementary Figure S1). In addition, performing physical exercise regularly with a vigorous exercise routine was acknowledged as regular physical exercise (high-intensity exercise ≥ 3 times/week or moderate-intensity exercise ≥ 5 times/week) [14].

Having a BMI ≥ 25 kg/m^2^ is defined as obesity according to the Asia-Pacific region criteria [15]; type 2 diabetes mellitus is defined by the International Classification of Disease, 10th Revision (ICD-10) codes E11–14 as either at least one prescription claim per year for antidiabetic treatment or a fasting glucose level of ≥7 mmol/L; hypertension is defined as having at least one prescription claim per year for an antihypertensive agent or a blood pressure of ≥140/90 mmHg under ICD-10 codes I10–I15 [16].

The primary endpoint was subsequently occurring CRC at least one year after the cohort entry (2011), defined with new ICD C18–20, and claims for deductions on medical treatment of malignant CRC (V codes, V193).

### 2.4. Ascertainment of Metabolic Syndrome and Change of Status

In the present study, MetS is defined by the International Diabetes Federation [17] to have at least three of the following five criteria: (a) high blood pressure (systolic blood pressure ≥ 130 mmHg, and diastolic blood pressure ≥ 85 mmHg), or medical treatments regarding previously diagnosed hypertension; (b) high triglyceride (TG) levels (≥150 mg/dL); (c) low HDL-C levels (<40 mg/dL for men and <50 mg/dL for women); (d) high levels of fasting plasma glucose (≥100 mg/dL) or previously diagnosed with type 2 diabetes; and (e) abdominal obesity (waist circumference ≥ 90 cm for men, ≥85 cm for women); abdominal obesity was revised based on the International Obesity Task Force Asia–Pacific region and the cut-offs for Korean adults [18].

Participants were classified into four groups according to changes in MetS status at the first and second examinations: MetS-never group and MetS-developed group (no MetS in 2009 but presence of MetS in 2011). Individuals having MetS at the first health examination were categorized into either the MetS-persistent group (presence of MetS in 2009 and 2011) or the MetS-resolved group (presence of MetS in 2009 but no MetS in 2011). Resolution of MetS is defined as a status with ≤2 of MetS components in this study regardless of medication [19].

### 2.5. Statistical Analyses

To compare baseline characteristics, one-way analysis of variance and chi-square tests were used for continuous and categorical variables, respectively. The Bonferroni method was used for multiple analyses (Supplementary Table S1). The cumulative incidence probabilities of CRC were plotted using Kaplan–Meier curves and compared using the log-rank test. To determine the independent association between MetS and the risk of CRC, we utilized the Cox proportional hazards model, which was modified for age, sex, smoking status, alcohol consumption level, and physical exercise habit, collected in 2011. It was assumed that the lifestyle in 2011 was continued to the end of follow-up and set the baseline covariates.

All statistical analyses were conducted utilizing the R version 3.2.3 (The R Foundation for Statistical Computing, Vienna, Austria, http://www.Rproject.org) (accessed on 20 July 2023), SAS version 9.4 (SAS Institute, Cary, NC, USA), and a two-sided *p*-value < 0.05 was regarded as statistically meaningful.

## 3. Results

### 3.1. Sociodemographic Characteristics

Table 1 displays the initial characteristics of the four MetS status groups. The MetS-free group had the highest proportion of persons who exercised regularly and the lowest proportion of heavy alcohol drinkers (all *p* < 0.001). The MetS-resolved and -developed groups were similar: they were likely to be older and consume excessive alcohol but less likely to exercise regularly than the MetS-free group (all *p* < 0.001). The MetS-resolved group included a higher proportion of participants who exercised more frequently than the others (*p* < 0.001). The MetS-persistent group was the oldest and included the lowest proportion of individuals who exercised regularly but the highest proportion of the bottom 20% of income earners (all *p* < 0.001).

### 3.2. Changes in the Status of Metabolic Syndrome and CRC Development

Among the 3,481,564 MetS-never participants, a total of 7318 (0.21%) developed CRC; in contrast, of the 760,929 persons who newly developed MetS, 2535 (0.33%) were diagnosed with CRC (Table 2). Among the 432,190 MetS-resolved participants, 1616 (0.37%) developed CRC during the follow-up period. Finally, of the 1,029,928 MetS-persistent participants, 4985 (0.48%) were diagnosed with CRC (Table 2).

**Figure 2:**
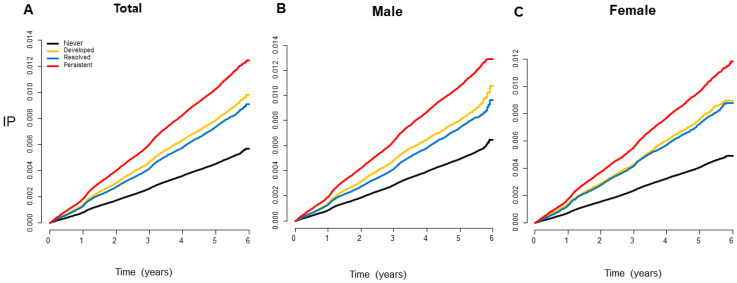
Kaplan–Meier curves showing the incidence probabilities of colorectal cancer according to changes in MetS status. The incidence probability of colorectal cancer was consistently lower in the MetS-recovered group than that in the MetS-persistent group and was higher in the MetS-developed group than that in the MetS-free group, regardless of sex (all log-rank *p* < 0.001). IP, incidence probability.

**Figure 3:**
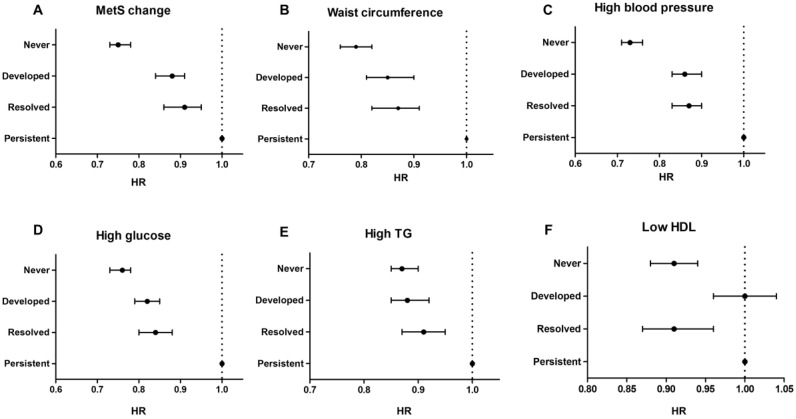
Colorectal cancer risks between the groups according to the change of metabolic syndrome status (**A**) and its individual factors (**B**–**F**). In this forest plot, persistent groups are used as a reference to emphasize that remission or improvement in metabolic syndrome can reduce the risk of colorectal cancer.

Kaplan–Meier curves presented that the incidence probability of CRC was higher in the MetS-developed group than in the MetS-free group, continuously, and it was consistently lower in the MetS-recovered group than in the MetS-persistent group, regardless of sex (Figure 2) (all *p* for log-rank test < 0.001). Multivariate analyses showed that the MetS-developed, -resolved, and -persistent groups had 17%, 24%, and 36% increases in the CRC risk compared to the MetS-never group, respectively (Table 2). When the MetS-persistent group was set as a reference, the hazard ratios (HRs) for CRC development in the MetS-free and MetS-developed were 0.75 (95% confidence interval (CI), 0.73–0.78) and 0.88 (95% CI, 0.84–0.91), respectively (Supplementary Table S2). The enrollees who recovered from MetS had a higher risk of CRC than the MetS-never individuals, but they had a lower risk than those who with MetS persistently (0.91 (95% CI, 0.86–0.95) vs. 0.75 (95% CI, 0.73–0.78)) (*p* < 0.001) (Figure 3A). When the results were stratified by sex, the CRC risks were not changed much (Supplementary Table S2).

### 3.3. Changes in Individual MetS Components and CRC Risk

We investigated whether the changes in MetS components are related to the CRC risk (Table 2). Compared to having no abnormal MetS components in both 2009 and 2011 health examinations, having abnormal values beyond the MetS threshold of any of the five factors was associated with an increased risk for the development of CRC (Table 2).

Recovery from each MetS component was related to a higher risk of CRC than that in never-MetS component or newly having MetS component, but recovery of each MetS component was associated with a lower risk of CRC than that associated with MetS-persistent component (*p* < 0.001) (Figure 3B–D).

**Figure 4:**
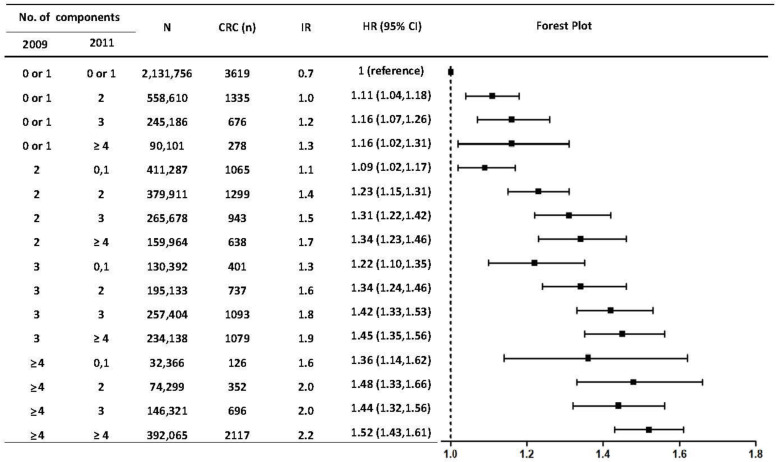
Risks of developing colorectal cancer according to the group in which the number of metabolic syndrome factors was changed. IR: per 1000 person-years. Hazard ratios were adjusted for age, sex, smoking, alcohol consumption, and regular exercise habit at the baseline examination (2011). F/U, follow-up duration; IR, incidence rate; HR, hazard ratio; CI, confidence interval; MetS, metabolic syndrome.

When stratification was conducted by sex, correcting abnormal waist circumference and fasting glucose components had a consistently preventive CRC effect compared to having each MetS component persistently, regardless of sex (Supplementary Table S2). The remission of high blood pressure and hypertriglyceridemia was related to a decreased risk for CRC among men but not in women, while resolving low HDL levels was seen among women but not in men (Supplementary Table S2). Furthermore, the risk for CRC development increased sequentially as more MetS components were developed at the point of registration (2011) compared to the previous examination (2009), when not having any components in the biennial exams was set as a reference (Figure 4).

### 3.4. Joint Effect of Resolving Abdominal Obesity and Modifying Other MetS Components on Developing CRC

Abdominal obesity is the predominant risk factor for metabolic syndrome and the first modifiable factor through lifestyle adjustment. Further analyses were performed to find out whether there is a synergistic effect on CRC prevention when another factor is resolved, in addition to the remission of abdominal obesity. Supplementary Table S2 shows that when the resolving of abnormal blood pressure or glucose metabolism is added by the remission of abdominal obesity, an addictive effect was generally observed. However, the recovery of TG and HDL-C levels to within the normal threshold did not show any CRC preventive effect without the remission of abdominal obesity (consistent abdominal obesity and unchanged high TG vs. consistent abdominal obesity but changed to normal TG: 1.32 (95% CI, 1.26–1.39) vs. 1.32 (95% CI, 1.21–1.44); consistent abdominal obesity and persistent low HDL-C vs. consistent abdominal obesity and changed to normal HDL-C: 1.30 (95% CI, 1.23–1.38) vs. 1.32 (95% CI, 1.20–1.45)). When both abdominal obesity and abnormal HDL were resolved together, the risk of CRC was reduced by 30% compared to the reference group (HR 0.71; 95% CI 0.61–0.81) (Supplementary Table S3).

## 4. Discussion

Through this longitudinal nationwide study of >5 million Korean participants, we observed that resolving MetS or its components results in an approximately 10% decreased risk of CRC compared to the status of the persistent-MetS. This study found a linear trend between the number of resolved MetS components and decreased CRC risk. In particular, normalizing waist circumference, fasting blood sugar, and blood pressure, even before being diagnosed with overt hypertension or diabetes, can reduce the risk of CRC. In addition, our data showed that MetS-developed individuals had a higher risk of CRC compared to non-MetS individuals at two consecutive exams, which supported the association between MetS and CRC development.

Although numerous research has evaluated the relationship between the presence or absence of MetS and the neoplasm of the colon [9,10,12,13], most of them are cross-sectional in nature, and anthropometric information is dependent on the patient’s recollections. To our knowledge, the present study is the first epidemiological study that has evaluated whether recovery from MetS status or its components could affect the risk of CRC by measuring MetS components at two time points.

Two studies have shown the preventive effect of keeping the waist–hip ratio within the normal range on CRC development, causing approximately 13% of case reduction [21,22]. Conversely, no remarkable increase in CRC risk has been reported in those who experienced an increased waist circumference [23]. As a study evaluating the change of variates in the time sequence, an evaluation of whether intentional weight reduction can lower the risk of cancer development has been carried out, but the result is still controversial [24,25]. Sensitivity analysis adjusting for other factors and BMI displayed equivalent results in the present study (Supplementary Table S4). More studies have shown that visceral fat or abdominal obesity are more closely related to MetS and CRC than subcutaneous fat obesity [26], which may be why CRC and MetS are consistently associated, regardless of BMI.

The potential preventive effect of glucose control on carcinogenesis could be inferred from the correlation between the glycemic index and CRC risk [27]. Upregulation of GLUT1 was reported in several malignancies, including CRC, and is one of the candidate pathways which accelerate the growth of CRC in the setting of hyperglycemia [28]. Moreover, chronic hyperglycemia contributes to hyperinsulinemia and insulin resistance; this activates the insulin growth factor signaling the pathway, which is the most adopted mechanism that accounts for the relationship between hyperglycemia and elevated cancer risk [29]. Since insulin receptors are widely expressed in CRC cells, controlling hyperglycemia can reduce hyperinsulinemia and may, in turn, hamper induction of EMT, insulin/IGF-1, and PI3K-AKT-mTOR signals, leading to continuous survival and division of CRC [25].

The cancer prevention mechanisms of lipid metabolism and blood pressure regulation have not been studied further compared to other MetS components. Considering that the sole improvement of lipid profile did not have a remarkable CRC-preventive effect unless the abdominal obesity was resolved in the present study, resolution of abdominal obesity may be more essential than lipid control. Conversely, the participants resolving both abdominal obesity and low HDL-C level were far less likely to develop CRC compared to those with persistent low HDL-C. Further study is required to elucidate the characteristics and cancer-preventive mechanism of this population.

This study had several limitations. First, changes in MetS status at initial classification may not be sustainable during the follow-up. CRC risk increases in a dose-dependent manner with the number of MetS diagnoses at two time points may suggest that quantifying the duration of MetS disease may be more realistic (Figure 2).

Second, the duration from MetS diagnosis to CRC development is somewhat short. Third, although the outcome was adjusted for age, sex, smoking, alcohol consumption, and regular exercise (or BMI), there are still uncontrolled confounding factors, including family history, medication history, and diet. In terms of drug history, some participants might have gained remission after antidiabetic agents, although our previous study showed that the majority of participants with MetS (75%) did not have diabetes [16]. Therefore, the present study needs to be interpreted cautiously due to the possibility of residual confounding bias.

Nevertheless, the strength of this research is that by assessing MetS status at two time points, we have shown that MetS can be changed reversibly, affecting the CRC risk. The research was based on the largest nationwide database available, encompassing nearly the whole population of South Korea, where both CRC and MetS are rapidly increasing. This study is of clinical importance because it provides insight that the heightened risk of CRC can be reduced through active control of MetS. Future studies are necessary to evaluate the impact of MetS remission on CRC risk with an adjustment of medication and a correction of MetS status, which may change over time.

## Figures and Tables

**Table 1:** Characteristics of the 5,704,611 enrollees of NHIC cohort, South Korea, 2011.

Variables	MetS-Never	MetS-Resolved	MetS-Developed	MetS-Persistent	*p* ^a^
	(n = 3,481,564)	(n = 432,190)	(n = 760,929)	(n = 1,029,928)	
Male sex	2,030,158 (58.3)	277,547 (64.2)	468,121 (61.5)	559,480 (54.3)	
Age (years)	46.7 ± 12.2	54.0 ± 12.6	53.3 ± 12.6	58.7 ± 12.1	<0.0001
Weight (kg)	62.8 ± 10.8	67.1 ± 11.8	68.5 ± 12.4	69.3 ± 12.9	<0.0001
Height (cm)	165.2 ± 8.9	164.4 ± 9.6	164.2 ± 9.7	162.3 ± 9.9	<0.0001
BMI (kg/m^2^)	22.9 ± 2.8	24.7 ± 2.9	25.3 ± 3.0	26.1 ± 3.2	<0.0001
Smoking					<0.0001
Non	2,016,806 (58.0)	228,588 (52.9)	410,693 (54.0)	612,036 (59.4)	
Ex	600,229 (17.3)	89,644 (20.8)	154,028 (20.3)	199,694 (19.4)	
Current	862,728 (24.8)	113,762 (26.3)	195,971 (25.8)	217,863 (21.2)	
Alcohol consumption ^b^					<0.0001
Non	1,634,606 (47.0)	214,836 (49.8)	380,433 (50.1)	599,638 (58.3)	
Mild to moderate	1,647,145 (47.4)	184,992 (42.9)	321,387 (42.3)	358,830 (34.9)	
Heavy (≥30 g/day)	193,929 (5.6)	31,827 (7.4)	58,138 (7.7)	70,284 (6.8)	
Regular exercise ^c^	2,114,649 (60.8)	251,896 (58.3)	429,640 (56.5)	538,646 (52.3)	<0.0001
Lowest quintile of income	671,600 (19.3)	93,037 (21.5)	166,377 (21.9)	238,905 (23.2)	
WC (cm)	78.0 ± 8.0	83.0 ± 7.6	85.4 ± 7.9	87.6 ± 8.2	<0.0001
Systolic BP (mmHg)	118.4 ± 13.0	124.3 ± 13.3	128.7 ± 13.4	130.1 ± 14.1	<0.0001
Diastolic BP (mmHg)	74.2 ± 9.1	77.4 ± 9.2	80.1 ± 9.5	79.8 ± 9.8	<0.0001
Fasting glucose (mg/dL)	92.8 ± 14.1	99.4 ± 23.0	104.9 ± 2 2.7	114.00 ± 32.7	<0.0001
Triglycerides ^d^ (mg/dL)	95.2 (95.2–95.3)	152.4 (152.2–152.5)	122.1 (122.0–122.3)	159.5 (159.3–159.6)	<0.001
HDL-cholesterol (mg/dL)	58 ± 14.1	52 ± 13.8	50 ± 14.0	49 ± 13.9	

Most of the data are presented as mean ± SD or number (percentage). Metabolic syndrome components: elevated waist circumference ≥ 90 cm for men and ≥85 cm for women; elevated blood pressure systolic ≥ 130 and/or diastolic ≥85 mmHg or use of antihypertensive medication; elevated fasting glucose ≥ 100 mg/dL or use of hypoglycemic agents; elevated serum triglycerides ≥ 150 mg/dL or use of lipid-lowering medication; reduced serum HDL-C < 40 mg/dL for men and <50 mg/dL for women or use of lipid-lowering medication. ^a^ Baseline characteristic was compared using a one-way analysis of variance for continuous variables and chi-square tests for categorical variables with Bonferroni correction. All groups are significantly different from each other in all variables except for the lowest quintile of income between MetS-developed and -resolved groups. The results of the Bonferroni correction are presented in Supplementary Table S1. ^b^ Mild-to-moderate alcohol consumption is defined as <30 g of alcohol/day, and heavy alcohol consumption is defined as ≥30 g of alcohol/day [20]. ^c^ Regular exercise is defined as intense 20 min workouts ≥3 days weekly or moderate 30 min workouts ≥5 days weekly. ^d^ Geometric means (95% CI). BMI, body mass index; BP, blood pressure; CRC, colorectal cancer; MetS, metabolic syndrome; NHIC, National Health Insurance Corporation; WC, waist circumference; HDL, high-density lipoprotein.

**Table 2:** Multivariate analyses for colorectal cancer risk according to changes of the individual element constituting metabolic syndrome.

Change	N	CRC	F/U Duration ^a^	IR	HR ^b^ (95% CI)
Metabolic syndrome					
Never	3,481,564	7318	8,332,787	0.88	1 (Reference)
Developed	760,929	2535	1,829,845	1.39	1.17 (1.11, 1.22)
Resolved	432,190	1616	1,043,387	1.55	1.24 (1.18, 1.31)
Persistent	1,029,928	4985	2,503,339	1.99	1.36 (1.30, 1.41)
Abnormal waist circumference ^c^					
Never	4,130,496	10,396	9,916,033	1.05	1 (Reference)
Developed	504,710	1532	1,212,377	1.26	1.08 (1.02, 1.14)
Resolved	366,171	1474	885,473	1.66	1.13 (1.07, 1.20)
Persistent	703,234	3052	1,695,474	1.80	1.32 (1.26, 1.40)
Abnormal blood pressure ^c^					
Never	2,440,459	4387	5,834,734	0.75	1 (Reference)
Developed	813,867	2197	1,954,729	1.12	1.14 (1.08, 1.20)
Resolved	632,632	1687	1,518,393	1.11	1.18 (1.11, 1.24)
Persistent	1,817,653	8183	4,401,502	1.86	1.36 (1.31, 1.42)
Abnormal fasting glucose ^c^					
Never	3,065,966	6723	7,363,893	0.91	1 (Reference)
Developed	904,834	2802	2,174,012	1.29	1.07 (1.02, 1.11)
Resolved	638,547	1888	1,531,460	1.23	1.11 (1.06, 1.17)
Persistent	1,095,264	5041	2,639,993	1.91	1.32 (1.27, 1.37)
Abnormal TG criterion ^c^					
Never	2,844,681	6747	6,818,793	0.99	1 (Reference)
Developed	852,405	2471	2,048,462	1.21	1.00 (0.96, 1.05)
Resolved	591,574	1935	1,428,350	1.35	1.02 (0.91, 1.08)
Persistent	1,415,951	5301	3,413,753	1.55	1.13 (1.09, 1.18)
Abnormal HDL cholesterol ^c^					
Never	3,356,868	7942	8,019,166	0.99	1 (Reference)
Developed	826,033	2836	1,990,435	1.42	1.09 (1.04, 1.13)
Resolved	552,854	1693	1,336,252	1.27	1.00 (0.95, 1.06)
Persistent	968,856	3983	2,363,504	1.69	1.10 (1.05, 1.14)

^a^ per 1000 person-years. ^b^ Adjusted for age, sex, smoking, alcohol consumption, and regular exercise habit at the 2011’s examination. ^c^ Based on the definition of metabolic syndrome, obesity is defined as a body mass index ≥25 kg/m^2^; diabetes mellitus is defined as fasting glucose levels ≥126 mg/dL measured during medical screening or antidiabetic drug prescriptions in conjunction with ICD-10-CM codes E11–E14; dyslipidemia is defined as total cholesterol levels ≥240 mg/dL or ICD-10-CM code E78 and claims for lipid-lowering agents; hypertension is defined as systolic blood pressure ≥140 mmHg, diastolic blood pressure ≥ 90 mmHg, or ICD-10-CM codes I10–I13 or I15 and claims for antihypertensive agents. BP, blood pressure; HDL-C, high-density lipoprotein cholesterol; ICD-10-CM, International Classification of Diseases, 10th Revision, Clinical Modification; LDL-C, low-density lipoprotein; F/U, follow-up duration; IR, incidence rate; HR, hazard ratio; CI, confidence interval; TG, triglyceride.

## Data Availability

The datasets generated during and/or analyzed during the current study are available from the corresponding author upon reasonable request.

## Supplementary Materials

The following supporting information can be downloaded at: https://www.mdpi.com/article/10.3390/jcm12154889/s1, Table S1: Multiple comparison results related to Table 1 using the Bonferroni method; Table S2: Hazard ratios and 95% confidence intervals of colorectal cancer occurrence according to the change of metabolic syndrome status and its components; Table S3: Hazard ratios and 95% confidence intervals of colorectal cancer development according to the change of abdominal obesity and accompanied the other metabolic syndrome component; Table S4: Hazard ratios and 95% confidence intervals of colorectal cancer according to the change of metabolic syndrome status (body mass index was co-operated into covariates); Figure S1: Self-report questionnaires collected at every health check-up (provided by National Health Insurance Corporation of South Korea).
