# Supplementary material for: Alteration of Metabolic Syndrome Is Associated with the Decreased Risk of Colorectal Cancer
Source: J Clin Med. 2023 Jul 25;12(15):4889. doi: 10.3390/jcm12154889 (PMC10419554; doi:10.3390/jcm12154889)
Supplement: Supplementary file 1 [file jcm-12-04889-s001.zip › Supplementary table_S1_S4.pdf]

Supplementary Table S1. Multiple comparison results related to Table 1 using the Bonferroni method

	MetS-free vs. MetS-resolved	MetS-free vs. MetS-developed	MetS-free vs. MetS-persistent	MetS-resolved vs. MetS-developed	MetS-resolved vs. MetS-persistent	MetS-developed vs. MetS-persistent
Sex, male	<.0001	<.0001	<.0001	<.0001	<.0001	<.0001
Age	<.0001	<.0001	<.0001	<.0001	<.0001	<.0001
Weight	<.0001	<.0001	<.0001	<.0001	<.0001	<.0001
Height	<.0001	<.0001	<.0001	<.0001	<.0001	<.0001
BMI	<.0001	<.0001	<.0001	<.0001	<.0001	<.0001
Total cholesterol	<.0001	<.0001	<.0001	<.0001	<.0001	<.0001
Smoking	<.0001	<.0001	<.0001	<.0001	<.0001	<.0001
Drink	<.0001	<.0001	<.0001	<.0001	<.0001	<.0001
Exercise	<.0001	<.0001	<.0001	<.0001	<.0001	<.0001
Income	<.0001	<.0001	<.0001	1	<.0001	<.0001
Waist circum.	<.0001	<.0001	<.0001	<.0001	<.0001	<.0001
SBP	<.0001	<.0001	<.0001	<.0001	<.0001	<.0001
DBP	<.0001	<.0001	<.0001	<.0001	<.0001	<.0001
Glucose	<.0001	<.0001	<.0001	<.0001	<.0001	<.0001
TG	<.0001	<.0001	<.0001	<.0001	<.0001	<.0001
HDL	<.0001	<.0001	<.0001	<.0001	<.0001	<.0001

Supplementary Table S2. Hazard ratios and 95% confidence intervals of colorectal cancer occurrence according to the change of metabolic syndrome status and its components

Change	N	CRC	F/U duration [a]	IR	HR (95% CI)		
					Total	Male	Female
Metabolic syndrome							
Never	3,481,564	7,318	8,332,787	0.88	0.75(0.73, 0.78)	0.67(0.64, 0.69)	0.83(0.79, 0.88)
Developed	760,929	2,535	1,829,845	1.39	0.88(0.84, 0.91)	0.84(0.79, 0.88)	0.90(0.85, 0.96)
Resolved	432,190	1,616	1,043,387	1.55	0.91(0.86, 0.95)	0.89(0.84, 0.95)	0.87(0.81, 0.95)
Persistent	1,029,928	4985	2,503,339	1.99	1 [Reference]	1 [Reference]	1 [Reference]
Abnormal waist circumference[c]							
Never	4,130,496	10,396	9,916,033	1.05	0.79(0.76, 0.82)	0.72(0.69, 0.75)	0.86(0.81, 0.91)
Developed	504,710	1,532	1,212,377	1.26	0.85 (0.81,0.90)	0.81(0.76, 0.86)	0.93(0.85, 1.01)
Resolved	366,171	1,474	885,473	1.66	0.87(0.82, 0.91)	0.90(0.84, 0.96)	0.90 (0.83, 0.98)
Persistent	703,234	3,052	1,695,474	1.80	1 [Reference]	1 [Reference]	1 [Reference]
Abnormal blood pressure[c]							
Never	2,440,459	4,387	5,834,734	0.75	0.73(0.71, 0.76)	0.62(0.60, 0.65)	0.84 (0.80, 0.89)
Developed	813,867	2,197	1,954,729	1.12	0.86(0.83, 0.90)	0.82(0.78, 0.86)	0.93(0.87, 0.99)
Resolved	632,632	1,687	1,518,393	1.11	0.87(0.83, 0.90)	0.82(0.78, 0.86)	0.95 (0.88, 1.03)
Persistent	1,817,653	8,183	4,401,502	1.86	1 [Reference]	1 [Reference]	1 [Reference]
Abnormal fasting glucose[c]							
Never	3,065,966	6,723	7,363,893	0.91	0.76(0.73, 0.78)	0.70(0.67, 0.73)	0.84(0.79, 0.88)
Developed	904,834	2,802	2,174,012	1.29	0.82(0.79, 0.85)	0.77(0.73, 0.81)	0.92(0.86, 0.98)
Resolved	638,547	1,888	1,531,460	1.23	0.84(0.80, 0.88)	0.81(0.76, 0.85)	0.90(0.83, 0.97)
Persistent	1,095,264	5,041	2,639,993	1.91	1 [Reference]	1 [Reference]	1 [Reference]
Abnormal TG criterion[c]							
Never	2,844,681	6,747	6,818,793	0.99	0.87(0.85, 0.90)	0.84(0.81, 0.87)	0.89(0.84, 0.93)
Developed	852,,405	2,471	2,048,462	1.21	0.88(0.85, 0.92)	0.86(0.82, 0.90)	0.91(0.85, 0.97)
Resolved	591574	1,935	1,428,350	1.35	0.91(0.87, 0.95)	0.82(0.78, 0.87)	0.96 (0.89, 1.04)

Persistent	1415951	5,301	3,413,753	1.55	1 [Reference]	1 [Reference]	1 [Reference]
Abnormal HDL cholesterol[c]							
Never	3,356,868	7,942	8,019,166	0.99	0.91(0.88, 0.94)	0.91(0.87, 0.96)	0.92(0.88, 0.97)
Developed	826,033	2,836	1,990,435	1.42	1.00(0.96, 1.04)	1.06(1.01, 1.13)	0.94(0.88, 1.00)
Resolved	552,854	1,693	1,336,252	1.27	0.91(0.87, 0.96)	1.02(0.96, 1.09)	0.87(0.81, 0.93)
Persistent	968,856	3,983	2,363,504	1.69	1 [Reference]	1 [Reference]	1 [Reference]

[a] per 1000 person-years.

[b] Adjusted for age, sex, smoking, alcohol consumption and regular exercise habit at the 2011's examination

[c] Based on the definition of metabolic syndrome; Obesity was defined as a body mass index ≥ 25 kg/m2; diabetes mellitus was defined as fasting glucose levels
≥ 126 mg/dL measured during medical screening or antidiabetic drug prescriptions in conjunction with ICD-10-CM codes E11–E14; dyslipidemia was defined as total cholesterol levels ≥ 240 mg/dL or ICD-10-CM code E78 and claims for lipid-lowering agents; hypertension was defined as systolic blood pressure ≥ 140 mmHg, diastolic blood pressure ≥ 90 mmHg, or ICD-10-CM codes I10–I13 or I15 and claims for antihypertensive agents.

BP, blood pressure; HDL-C, high-density lipoprotein cholesterol; ICD-10-CM, International Classification of
Diseases, 10th Revision, Clinical Modification; LDL-C, low-density lipoprotein; F/U, Follow-up duration; IR, incidence rate; HR, hazard ratio; CI, confidence interval; TG, triglyceride

Supplementary Table S3. Hazard ratios and 95% confidence intervals of colorectal cancer development according to the change of abdominal obesity and accompanied the other metabolic syndrome component

Abdominal obesity	Second component	High blood pressure			High blood glucose			High TG			Low HDL		
		CRC	IR[b]	HR[c] (95% CI)	CRC	IR[b]	HR[c] (95% CI)	CRC	IR[b]	HR[c] (95% CI)	CRC	IR[b]	HR[c] (95% CI)
Free	Free	5090	0.73	1 [Reference]	6961	0.85	1 [Reference]	7224	0.90	1 [Reference]	8267	0.92	1 [Reference]
	Developed	2125	1.10	1.13(1.08, 1.19)	2523	1.21	1.10(1.05, 1.15)	2205	1.12	1.01(0.97, 1.06)	2369	1.30	1.09(1.04, 1.14)
	Resolved	1660	1.06	1.14(1.08, 1.21)	1720	1.10	1.08(1.02, 1.13)	1728	1.26	1.02(0.97, 1.07)	1493	1.14	1.06(1.00, 1.12)
	Persistent	6101	1.72	1.30(1.25, 1.35)	3772	1.75	1.31(1.26, 1.36)	3819	1.43	1.14(1.09, 1.18)	2847	1.49	1.06(1.02, 1.11)
Developed	Free	429	0.82	1.04(0.95, 1.15)	854	1.06	1.10(1.02, 1.18)	778	1.27	1.19(1.11, 1.28)	927	1.09	1.08(1.01, 1.16)
	Developed	358	1.16	1.19(1.07, 1.33)	456	1.35	1.21(1.10, 1.33)	405	1.12	1.01(0.91, 1.11)	457	1.41	1.12(1.02, 1.23)
	Resolved	194	1.09	1.18(1.02, 1.36)	260	1.40	1.25(1.10, 1.41)	224	1.39	1.08(0.95, 1.24)	220	1.36	1.08(0.95, 1.24)
	Persistent	1276	1.82	1.35(1.27, 1.43)	687	1.78	1.25(1.16, 1.36)	850	1.47	1.14(1.06, 1.22)	653	1.73	1.18(1.09, 1.28)
Resolved	Free	341	1.00	0.85(0.76, 0.95)	680	1.30	1.11(1.03, 1.20)	636	1.52	0.99(0.91, 1.08)	840	1.49	1.22(1.13, 1.31)
	Developed	240	1.43	1.24(1.09, 1.41)	326	1.63	0.92(0.82, 1.04)	286	1.68	1.17(1.04, 1.31)	375	1.81	1.21(1.09, 1.34)
	Resolved	226	1.42	1.27(1.11, 1.45)	265	1.66	1.29(1.14, 1.46)	305	1.52	1.12(1.00, 1.26)	227	1.44	0.71(0.61, 0.81)
	Persistent	1275	2.19	1.44(1.35, 1.53)	811	2.20	1.43(1.33, 1.53)	855	1.85	1.27(1.18, 1.36)	640	1.98	1.21(1.12, 1.32)
Persistent	Free	478	1.11	1.18(1.08, 1.30)	1211	1.41	1.23(1.16, 1.31)	1101	1.79	1.36(1.27, 1.44)	1448	1.52	1.28(1.21, 1.35)
	Developed	523	1.49	1.39(1.27, 1.52)	748	1.63	1.31(1.21, 1.41)	650	1.67	1.23(1.13, 1.33)	894	1.95	1.36(1.27 1.46)
	Resolved	349	1.44	1.39(1.25, 1.55)	451	1.73	1.36(1.23, 1.49)	531	1.87	1.32(1.21, 1.44)	485	1.85	1.32(1.20, 1.45)
	Persistent	2979	2.18	1.53(1.46, 1.60)	1919	2.34	1.55(1.47, 1.63)	2047	1.85	1.32(1.26, 1.39)	1502	2.08	1.30(1.23, 1.38)

Column group heading: The second component[a]

IR, incidence rate; HR, hazard ratio; CI, confidence interval; TG, triglyceride; HDL, High-density lipoprotein

*The non-metabolic syndrome group in both 2009 and 2011 exam was set as a reference

[a] Based on the definition of metabolic syndrome

[b] per 1000 person-years

[c]Adjusted for age, sex, smoking, alcohol consumption and regular exercise habit at the examination of 2011(index date)

Supplementary Table S4. Hazard ratios and 95% confidence intervals of colorectal cancer according to the change of metabolic syndrome status (body mass index was cooperated into covariates)

	N	CRC (n)	Follow-up duration[a]	IR[b]	HR[c] (95% CI)
Metabolic syndrome					
Free	3481564	7318	8332787	0.88	1[Reference]
Developed	760929	2535	1829845	1.39	1.17(1.11,1.22)
Resolved	432190	1616	1043387	1.55	1.24(1.18,1.31)
Persistent	1029928	4985	2503339	1.99	1.36(1.30,1.41)
Waist circumference criterion					
Free	4130496	10396	9916033	1.05	1[Reference]
Developed	504710	1532	1212377	1.26	1.08(1.02,1.14)
Resolved	366171	1474	885473	1.66	1.13(1.07,1.20)
Persistent	703234	3052	1695474	1.80	1.32(1.26,1.40)
Blood pressure criterion					
Free	2440459	4387	5834734	0.75	1[Reference]
Developed	813867	2197	1954729	1.12	1.14(1.08,1.20)
Resolved	632632	1687	1518393	1.11	1.18(1.11,1.24)
Persistent	1817653	8183	4401502	1.86	1.36 (1.31,1.42)
Fasting glucose criterion					
Free	3065966	6723	7363893	0.91	1[Reference]
Developed	904834	2802	2174012	1.29	1.07(1.02,1.11)
Resolved	638547	1888	1531460	1.23	1.11(1.06,1.17)
Persistent	1095264	5041	2639993	1.91	1.32(1.27,1.37)
TG criterion					
Free	2844681	6747	6818793	0.99	1[Reference]
Developed	852405	2471	2048462	1.21	1.00(0.96,1.05)
Resolved	591574	1935	1428350	1.35	1.02(0.97,1.08)
Persistent	1415951	5301	3413753	1.55	1.13(1.09,1.18)
HDL cholesterol criterion					
Free	3356868	7942	8019166	0.99	1[Reference]
Developed	826033	2836	1990435	1.42	1.09(1.04,1.13)
Resolved	552854	1693	1336252	1.27	1.00(0.95,1.06)
Persistent	968856	3983	2363504	1.69	1.10(1.05,1.14)

The non-metabolic syndrome group in both 2009 and 2011 exam was set as a reference

[a] person-years

[b] Colorectal cancer incidence per 1000 person-years

c Adjusted for age, sex, smoking status, alcohol consumption, and regular physical exercise and body mass index
